# ﻿Evidence using morphology, molecules, and biogeography clarifies the taxonomic status of mole crabs of the genus *Emerita* Scopoli, 1777 (Anomura, Hippidae) and reveals a new species from the western Atlantic

**DOI:** 10.3897/zookeys.1161.99432

**Published:** 2023-05-12

**Authors:** Fernando L. Mantelatto, Juliana M. Paixão, Rafael Robles, Jeniffer N. Teles, Felipe C. Balbino

**Affiliations:** 1 Laboratory of Bioecology and Crustacean Systematics (LBSC), Faculty of Philosophy, Sciences and Letters at Ribeirão Preto (FFCLRP), University of São Paulo (USP), Av. Bandeirantes 3900, 14040-901, Ribeirão Preto, SP, Brazil University of São Paulo Ribeirão Preto Brazil; 2 Facultad de Ciencias Químico-Biológicas, Universidad Autónoma de Campeche, Campus V. Predio s/n – Avenida Ing. Humberto Lanz Cárdenas y Fracc. Ecológico Ambiental Siglo XXIII, Colonia Ex Hacienda Kalá, San Francisco de Campeche, Camp., 24085, Mexico Universidad Autónoma de Campeche Campeche Mexico

**Keywords:** 16S rRNA, COI, Cryptic diversity, distribution, molecular data, phylogeny

## Abstract

Uncertainties regarding the taxonomic status and biogeographical distribution of some species of the genus *Emerita* from the western Atlantic led to thorough examination of the subtle morphological differences between two coexistent species (*E.brasiliensis* Schmitt, 1935 and *E.portoricensis* Schmitt, 1935) along the Brazilian coast and compare them using two genetic markers. The molecular phylogenetic analysis based on sequences of the 16S rRNA and COI genes showed that individuals identified as *E.portoricensis* were clustered into two clades: one containing representatives from the Brazilian coast and another containing specimens distributed in Central America. Our molecular-based phylogeny, combined with a detailed morphological analysis, revealed the Brazilian population as a new species, which is described here as *Emeritaalmeidai* Mantelatto & Balbino, **sp. nov.** The number of species in the genus *Emerita* is now raised to 12, with five of them occurring in the western Atlantic, five in the Indo-Pacific, and two in the eastern Pacific.

## ﻿Introduction

The superfamily Hippoidea Latreille, 1825 is one of the seven superfamilies that belong to the highly diverse infraorder Anomura Macleay, 1838 (Boyko and McLaughlin 2010). It is represented by three families of sand/mole crabs: Albuneidae Stimpson, 1858 (nine genera and 53 recognized species), Blepharipodidae Boyko, 2002 (two genera and six species) and Hippidae Latreille, 1825 (three genera and 28 species) (Boyko 2002; WoRMS 2023). Representatives of all these families are well known due to their presence in intertidal sandy beaches of temperate, tropical, and subtropical areas. Their ability to bury themselves in this environment of constant hydrodynamics is one of the most outstanding characteristics of this group.

The genus *Emerita* Scopoli, 1777 (family Hippidae) contains eleven species that are widely distributed around the globe, living in intertidal and upper subtidal sandy marine regions. Their filter feeding habit is an ecologically essential activity in sandy beach environments (Rodgers 1987; Lercari and Defeo 1999; Hubbard and Dugan 2003). These sand crabs are also considered as bioindicators of environment quality (Pérez 2003; Petracco et al. 2003). Five species are reported in the Indo-Pacific [*Emeritaemeritus* (Linnaeus, 1767), *E.austroafricana* Schmitt, 1937, *E.holthuisi* Sankolli, 1965, *E.karachiensis* Niazi & Haque, 1974, and *Emeritataiwanensis* Hsueh, 2015], two in the eastern Pacific [*Emeritaanaloga* (Stimpson, 1857) and *Emeritarathbunae* Schmitt, 1935], and four in the western Atlantic [*Emeritatalpoida* Say, 1817, *E.benedicti* Schmitt, 1935, *E.brasiliensis* Schmitt, 1935, and *E.portoricensis* Schmitt, 1935].

Most of the studies that established the current taxonomic status of species in *Emerita* were based on morphology (see Calado 1990 and Melo 1999 for revisions). Molecular data has been used to test for genetic flow between distant populations of conspecifics of *E.analoga* and *E.talpoida* from the eastern Pacific and western Atlantic populations, respectively (Tam et al. 1996). Later, a molecular phylogeny, using two genetic markers, rejected the hypothesis that species of *Emerita* from the New World form a monophyletic group (Haye et al. 2002). Recently, specimens of *Emerita* from Indonesia were evaluated using DNA barcoding and morphology to identify, but not describe a new species (Bhagawati et al. 2020, 2022). More recently, the inaccurate identity of some specimens and the distribution of *E.portoricensis* was clarified (Felder et al. 2023).

The two species of *Emerita* reported from the Brazilian coast are *E.brasiliensis* and *E.portoricensis*. The former species can be found in Venezuela, Trinidad and Tobago, Brazil (Espírito Santo, Rio de Janeiro, São Paulo, Paraná, Santa Catarina, Rio Grande do Sul), Uruguay, and Argentina (Efford 1976; Calado 1998; Melo 1999; Veloso and Cardoso 1999; Spivak et al. 2019; Mantelatto et al. 2021), with a gap of records from Venezuela to Bahia (Brazil), the species is abundant from Espírito Santo to southern Brazil (Mantelatto et al. 2021; present work). *Emeritaportoricensis* occurs in Central American mainland shorelines of the Caribbean Sea, confirmed to include, but not limited to Puerto Rico, Dominican Republic, Virgin Islands, Jamaica, Belize, Costa Rica, Panama, Colombia, St. Lucia, St. Thomas, Venezuela, and Trinidad and Tobago (Felder et al. 2023); Brazilian records (from Maranhão to Sergipe) have been treated as *E.portoricensis* by several authors (Schmitt 1935; Efford 1976; Calado 1990; Melo 1999).

These patterns of geographical distribution and uncertain records in the western Atlantic raise questions about whether these gaps are due to a lack of faunal surveys and/or a misidentification of specimens that are morphologically similar. Thus, we were motivated to perform a reassessment of the specimens assigned as *E.portoricensis* and *E.brasiliensis* along the Brazilian coast, using both morphological and molecular tools to evaluate the phylogenetic relationships between species of *Emerita*. We also examined the possible existence of cryptic taxa, which resulted in the new species described herein.

## ﻿Materials and methods

### ﻿Sample collection and morphological data

Almost all specimens of *Emerita* analyzed herein were obtained by us and are deposited in the Crustacean Collection of the Department of Biology (**CCDB**) at the Faculty of Philosophy, Sciences and Letters at Ribeirão Preto (**FFCLRP**), University of São Paulo (**USP**), Brazil. Additional species of *Emerita* and other genera of Albuneidae (see Boyko 2002) were obtained and used in order to root the phylogenetic analyses. Individuals were collected by hand during low tide at different sandy beaches along the geographic distribution of the species (see references in Introduction). We also studied specimens obtained by means of loans or donations from University of Louisiana at Lafayette Zoological Collection, LA, United States (**ULLZ** – recently transferred to the National Museum of Natural History, Smithsonian Institution, Washington, D.C.**USNM**; old catalog numbers are used in the text).

**Figure 1.**
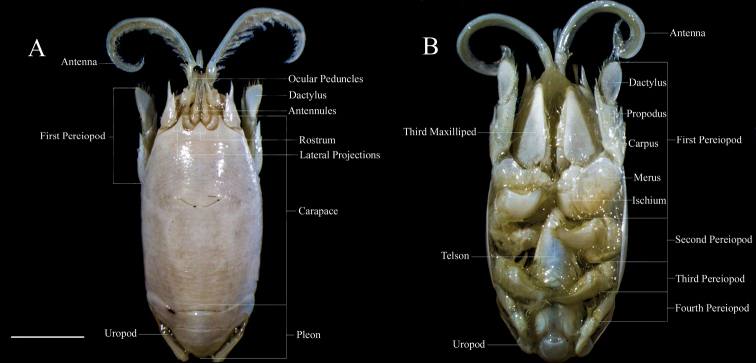
**A** dorsal view of adult ♀ of *Emeritaalmeidai* sp. nov. (CCDB 3369) **B** ventral view of adult ♀ of *Emeritabrasiliensis* (CCDB 2552). The main characters used for external morphology analysis are labeled. Scale bar: 5 mm.

Specimens were identified according to previous morphological characters established in the literature (Calado 1990; Melo 1999; Felder et al. 2023). All data along with new characters/variation were also considered for the comparative analysis along the species’ geographic distribution. When secondary sexual characters (presence of the gonopores on the coxae of the fifth pair of pereopods and absence of mature pleopods for males – ♂s, and the presence of the gonopores on the coxae of the third pair of pereopods and presence of mature pleopods or eggs for females – ♀s) were not conspicuously observed, specimens were classified as juveniles (Delgado and Defeo 2006). Most of the morphological characters followed the references cited above and are designated in Fig. 1. Analyses were made and photographs were taken under a LEICA M205C stereomicroscope equipped with a LEICA DFC 295 camera, and measurements (mm) of structures were taken using the software Leica Application Suite.

### ﻿Abbreviations

**coll(s).** collector(s),

**cl.** carapace length,

**cw.** carapace width,

**dl.** dactylus length,

**dw.** dactylus width,

**tl.** telson length,

**tw.** telson width.

### ﻿Brazilian states

**BA** Bahia,

**CE** Ceará,

**ES** Espírito Santo,

**PE** Pernambuco,

**RJ** Rio de Janeiro,

**RN** Rio Grande do Norte,

**SC** Santa Catarina,

**SP** São Paulo.

### ﻿Molecular data

The molecular markers 16S rRNA and cytochrome c oxidase subunit I (COI) were chosen because these mitochondrial genes are effective in studies that contribute to our comprehension of decapod diversity (see Schubart et al. 2000 and Timm and Bracken-Grissom 2015 for references), including anomuran members (Mantelatto et al. 2006, 2009; Miranda et al. 2020) and the target genus (Bhagawati et al. 2020, 2022). In this study, we used four different primers (see below).

We used muscle tissue from the telson or 3^rd^ pereopods for DNA extraction according to the protocols proposed by Mantelatto et al. (2007) and Robles et al. (2007), and some adaptations were made to suit our material using the manufacturer’s protocol of the salting-out method (Miller et al. 1988). The extracted DNA’s final concentration was measured using a spectrophotometer (NanoDrop 2000/2000c). Approximately 658 base pairs (bp) of the COI and 316 bp of the 16S rRNA genes were amplified using polymerase chain reactions (PCR) by thermal cycler (Veriti 96 Well Thermal Cycler Applied Biosystems). Fragments were amplified using the following thermal profiles: 16S rRNA – initial denaturing for 2 min at 94 °C; annealing for 40 cycles, 45 s at 94 °C, 45 sec at 46 °C and 1 min at 72 °C; final extension for 10 min at 72 °C; COI – initial denaturing for 2 min at 94 °C; annealing for 35 cycles, 30 sec at 94 °C, 30 sec at 50 °C, and 1 min at 72 °C; final extension for 7 min at 72 °C. We used the following primers: 16S-1472 (5’– AGA TAG AAA CCA ACC TGG – 3’) (Crandall and Fitzpatrick 1996) and 16SL (5’– CGC CTG TTT ATC AAA AAC AT – 3’) (Palumbi and Benzie 1991), HCO1-2198 (5’– TAA ACT TCA GGG TGA CCA AAA AAT CA – 3’) and LCO1-1490 (5’– GGT CAA CAA ATC ATA AAG ATA TTG – 3’) (Folmer et al. 1994). PCR products were observed in electrophoresis with 1.0% agarose gel and photographed with digital camera Olympus C-7070 on a UV transilluminators M20 UVP. Successful PCR products were purified using the SureClean Plus kit, following the manufacturer’s protocol. Purified samples were sent to the Department of Technology at the College of Agricultural and Veterinary Sciences (FCAV, Jaboticabal) at São Paulo State University (**UNESP**) for sequencing.

A consensus was reached between the forward and reverse sequences of each specimen in BioEdit v. 7.0.5 (Hall 2005), and unspecific readings were manually corrected when required. Primer regions and non-readable parts at the beginning of the sequences were omitted. All consensus sequences were deposited in GenBank (http://www.ncbi.nlm.nih.gov/genbank/).

The alignment of the consensus of all sequences used in the phylogeny was performed with MAFFT (Katoh and Standley 2006) in the software Geneious 2022.1 (Kearse et al. 2012). Three maximum likelihood (ML) phylogenetic analyses were performed using the IQ-TREE program (Miller et al. 2010), one with the COI gene, one with the16S rRNA gene, and one using a concatenated alignment. The evolutionary model that best fit the data was determined by IQ-TREE according to the Bayesian Information Criterion (BIC) (Luo et al. 2010) and used for tree inference. The branch support was evaluated by ultra-fast bootstrap with 2000 pseudoreplicates.

## ﻿Results

### ﻿Molecular data

We generated new sequences for 38 individuals from different localities: for 16S rRNA – 1 of *Emeritaanaloga*, 10 of *Emeritaalmeidai* sp. nov., 1 of *Emeritabenedicti*, 11 of *Emeritabrasiliensis*, 1 of *Emeritaportoricensis*, 3 of *Emeritarathbunae*, and 3 of *Emeritatalpoida*; for COI – 9 of *E.almeidai* sp. nov., 1 of *E.analoga*, 1 of *E.benedicti*, 15 of *Emeritabrasiliensis*, 2 of *E.portoricensis*, 2 of *E.rathbunae*, and 2 of *E.talpoida*. Additional sequences from GenBank were used to build a robust reconstruction (Table 1).

**Table 1.** Species of *Emerita* and *Lepidopa* used in the molecular analyses. CCDB: Coleção de Crustáceos do Departamento de Biologia, FFCLRP, USP, Brazil; ULLZ: University of Louisiana at Lafayette Zoological Collection, USA (recently transferred to National Museum of Natural History, Smithsonian Institution, Washington, D.C. (USNM)); (-) = data not available.

Species	Locality	Catalogue number	GenBank accession number	
			COI	16S
*E.analoga*	California, USA	–	–	AF246153
	California, USA	–	–	L43107
	California, USA	–	–	L43108
	California, USA	–	–	AF425322
	Oregon, USA	–	GU443297	–
	_	–	HQ341148	–
	_	–	HQ340917	–
	Calfuco, Chile	CCDB 4870	OQ679992	KP091505
	Algaborro, Chile	–	–	AF246154
*E.almeidai* sp. nov.	Rio Grande Norte, Brazil	CCDB 3369	KP091512	KP091493
	Rio Grande Norte, Brazil	CCDB 3376	KP091509	KP091491
	Rio Grande Norte, Brazil	CCDB 3380	KP091507	KP091489
	Rio Grande Norte, Brazil	CCDB 3393	KP091514	KP091496
	Pernambuco, Brazil	CCDB 4937	KP091515	KP091498
	Alagoas, Brazil	CCDB 4869	KP091516	KP091497
	Bahia, Brazil	CCDB 2606	–	KP091495
	Bahia, Brazil	CCDB 3026	KP091508	KP091490
	Bahia, Brazil	CCDB 4262	KP091513	KP091494
	Espírito Santo, Brazil	CCDB 3992	KP091510	KP091488
	Rio de Janeiro, Brazil	CCDB 4376	KP091511	KP091492
*E.benedicti*	Los Tuxtlas, Mexico	CCDB 4674	KP091525	KP091501
	Texas, USA	–	–	AF256155
	Texas, USA	–	–	L43109
*E.brasiliensis*	Espírito Santo, Brazil	CCDB 3990	KP091533	KP091477
	Espírito Santo, Brazil	CCDB 3994	KP091536	KP091481
	Rio de Janeiro, Brazil	CCDB 4119	KP091537	KP091482
	Rio de Janeiro, Brazil	CCDB 4935	KP091527	–
	São Paulo, Brazil	CCDB 1442	KP091530	KP091475
	São Paulo, Brazil	CCDB 1443	KP091531	–
	São Paulo, Brazil	CCDB 2552	–	KP091478
	São Paulo, Brazil	CCDB 2751	–	KP091483
	São Paulo, Brazil	CCDB 3923	KP091529	–
	São Paulo, Brazil	CCDB 3924	KP091532	KP091476
	São Paulo, Brazil	CCDB 4617	KP091538	KP091484
	Santa Catarina, Brazil	CCDB 4407	KP091534	KP091479
	Santa Catarina, Brazil	CCDB 4409	KP091535	KP091480
	Rio Grande Sul, Brazil	CCDB 4985	KP091526	–
	Rio Grande Sul, Brazil	CCDB 4986	KP091528	–
	Rio Grande Sul, Brazil	CCDB 3921	KP091539	–
	Fortaleza de Santa Teresa, Uruguay	–	–	L43110
	–	–	–	DQ079712
*E.emeritus*	Pondichvory, India	–	–	AF2461556
*E.holthuisi*	Dubai, United Arab Emirates	–	–	AF246157
*E.portoricensis*	Mayaguez, Puerto Rico	–	–	L43111
	Boca del Drago, Panama	CCDB 3525	KP091517	KP091486
	Boca del Drago, Panama	USNM 1546871 (= ULLZ 13325)	KP091519	–
*E.rathbunae*	–	–	–	JN800539
	Acapulco, Mexico	CCDB 1029	KP091523	KP091499
*E.talpoida*	Los Tuxtlas, Mexico	CCDB 4675	KP091521	KP091502
	South Carolina, USA	–	–	AF246150
	Massachusetts, USA	–	–	AF246151
	Massachusetts, USA	–	–	L43112
	Massachusetts, USA	–	–	L43113
	Florida, USA	ULLZ 13055	KP091522	KP091503
	Florida, USA	ULLZ 10144	–	KP091504
	Florida, USA	–	–	L43114
	Florida, USA	–	–	AF246152
*Lepidoparichmondi*	São Paulo, Brazil	CCDB 3920	KP091540	KP091506

### ﻿16S rRNA reconstruction

**Figure 2.**
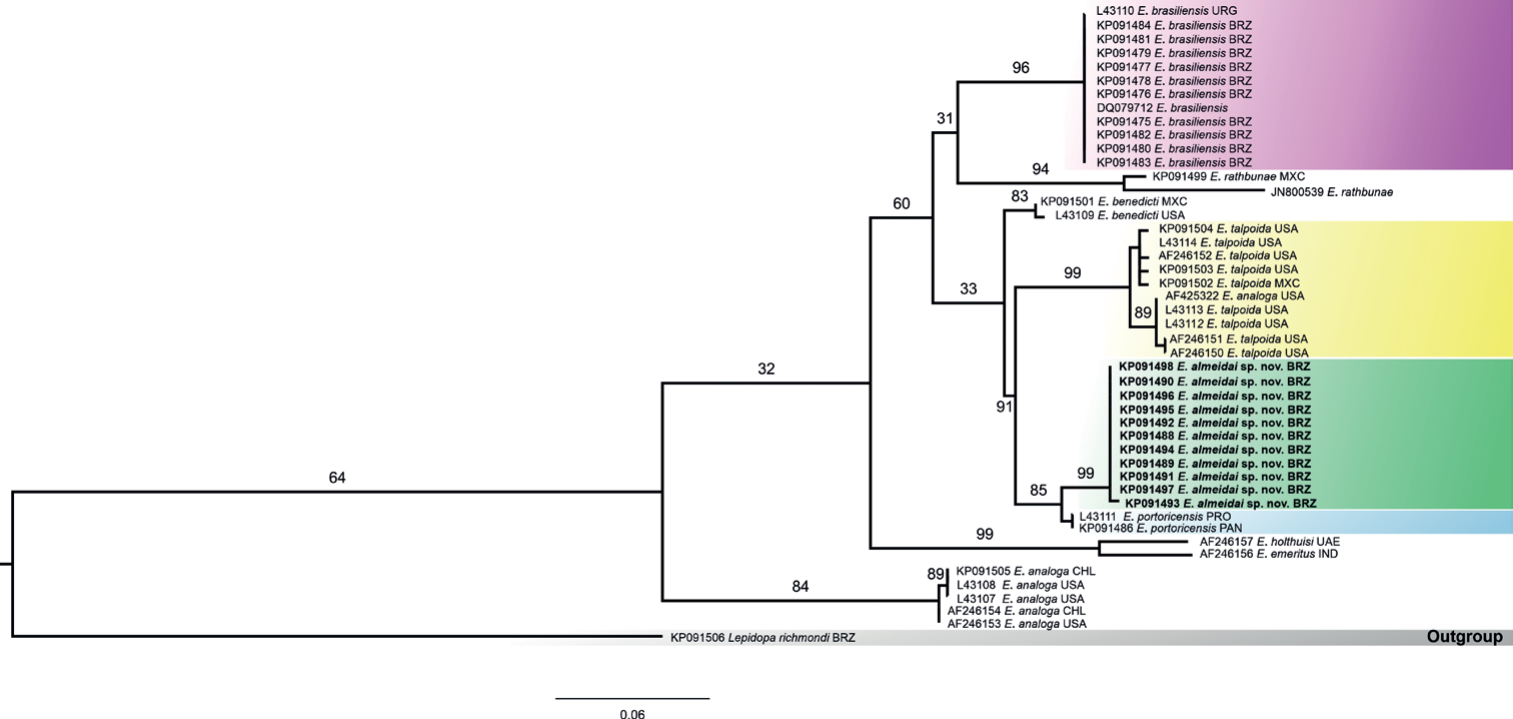
Maximum likelihood phylogram obtained for 16S rRNA sequences of *Emerita* specimens. Numbers represent bootstrap values (2000 pseudoreplicates). GenBank code is shown before the species name. Abbreviations: BRZ: Brazil; URG: Uruguay; MXC: Mexico; USA: United States of America; PAN: Panama; PRO: Puerto Rico; UAE: United Arab Emirates; IND: India; CHL: Chile.

The automated alignment of 16S rRNA with 316 bp included 50 sequences of *Emerita* species. The phylogenetic tree, generated by ML analyses, indicated a clear separation of each species of *Emerita* (Fig. 2). *Emeritabrasiliensis* consisted of a single clade, with all specimens assigned to this species, which was supported by bootstrap values of 96%. In this analysis, the closest relative of *E.brasiliensis* was *E.rathbunae*, although with low support (31%).

All specimens of *E.almeidai* sp. nov. were clustered in a strongly supported clade (bootstrap values of 91%), which was the sister group of *E.portoricensis* s.s. from Central America (bootstrap values of 99%). Specimens of *Emeritatalpoida* were split into two groups, one of them containing individuals from Florida (USA) and Mexico and the second one containing individuals from Massachusetts and South Carolina (USA). The positioning of a supposed “*E.analoga*” (AF425322) in this second group indicated a misidentification that should be fixed in the GenBank database. The phylogram positioned *E.benedicti* as a sister species of the clade composed by *E.almeidai* sp. nov., *E.portoricensis*, and *E.talpoida*, although with low support (60%). This major group, including *E.almeidai* sp. nov., *E.portoricensis*, *E.talpoida*, and *E.benedicti*, is the sister group of the clade composed of *E.brasiliensis* and *E.rathbunae*.

The clade containing *E.holthuisi* and *E.emeritus*, species from the Indo-Pacific, was positioned as a sister group of the major American clade mentioned above.

*Emeritaanaloga*, with reservations on the above-mentioned misidentified specimen, formed a single well-defined clade, with individuals from California (USA) and Chile, and was positioned as the sister species of all other species of *Emerita* used in the reconstruction, including members from the Americas as well as the Old World (*E.emeritus* and *E.holthuisi*).

### ﻿Cytochrome Oxidase I (COI) reconstruction

**Figure 3.**
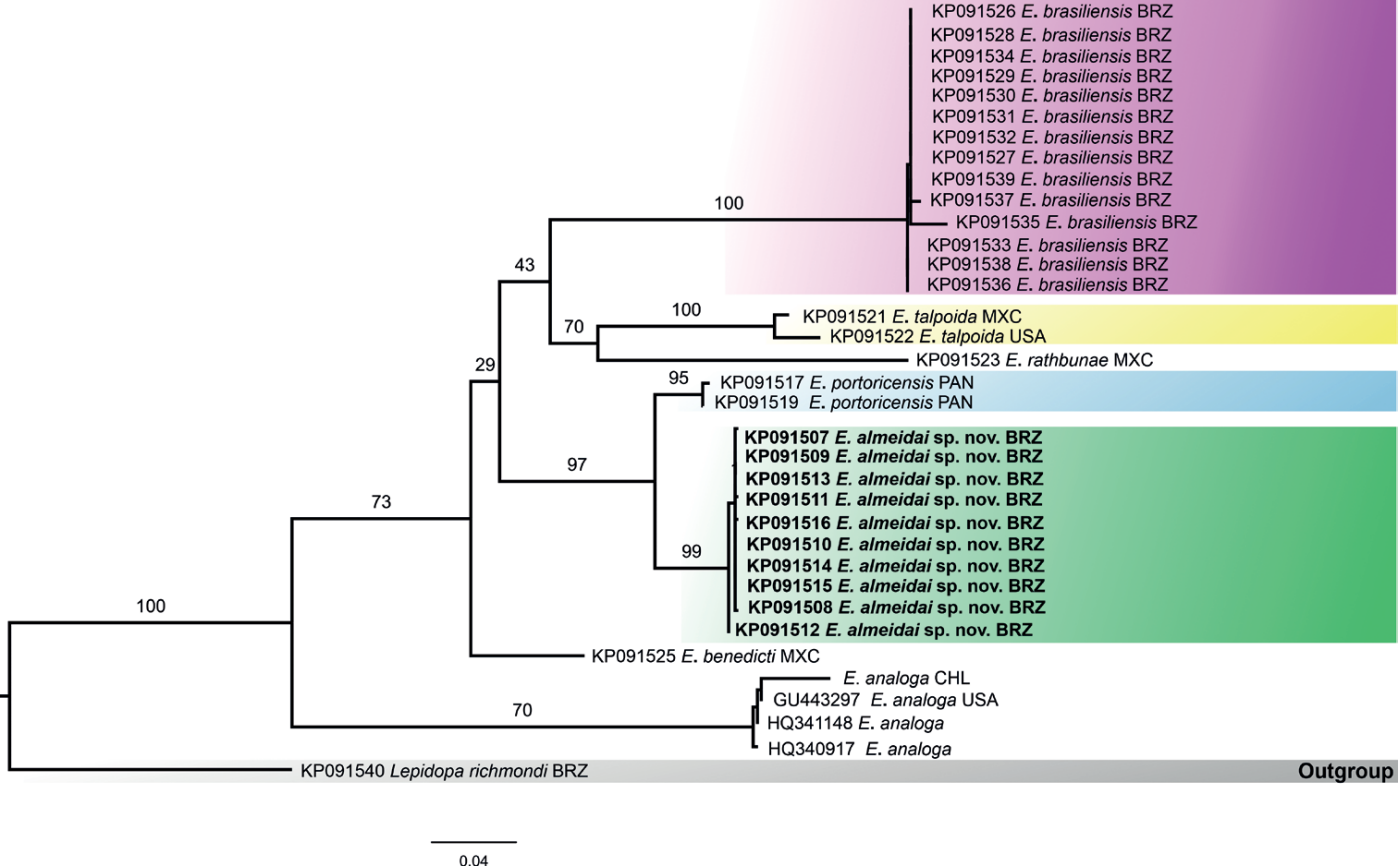
Maximum likelihood phylogram obtained for COI (HCO1/LCO1) sequences of *Emerita* specimens. Numbers represent bootstrap values (2000 pseudoreplicates). GenBank code is shown before the species name. Abbreviations: BRZ: Brazil; MXC: Mexico; USA: United States of America; PAN: Panama; CHL: Chile.

The automated alignment of COI sequences with 658 bp included some sequences of *Emerita* species from GenBank. The phylogram also confirmed the clear separation of every species of *Emerita* (Fig. 3), including the strongly supported position of *Emeritaalmeidai* sp. nov. Some differences were observed in the phylogenetic position of some of the species included in this alignment compared to that of the 16S rRNA alignment. For instance, *E.rathbunae* was recovered as the closest relative of *E.talpoida* instead of *E.brasiliensis*. The clade composed of *E.talpoida* and *E.rathbunae* was recovered as the sister group of *E.brasiliensis*. Furthermore, this clade [*E.brasiliensis* + (*E.rathbunae* + *E.talpoida*)] was recovered as the sister group of the clade composed of *E.portoricensis* and *E.almeidai* sp. nov. In this analysis, *E.benedicti* was found to be the sister species of the clade comprising *E.brasiliensis*, *E.talpoida*, *E.rathbunae*, *E.portoricensis*, and *E.almeidai* sp. nov.

Once again, despite the low number of specimens of *E.talpoida*, a clear division into two groups was recovered, with a few differences in relation to the 16S rRNA topology (the individual from Mexico was separated from the Florida one). The phylogenetic positioning of *E.analoga* was maintained as sister to all other species of *Emerita* included in this analysis.

### ﻿Concatenated phylogram

**Figure 4.**
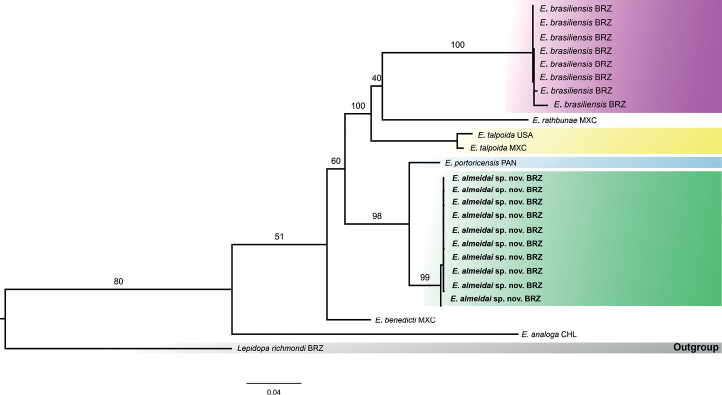
Concatenated tree molecular data set (16S rRNA and HCO1/LCO1) of maximum likelihood for *Emerita* specimens. Numbers represent bootstrap values (2000 replicates). GenBank code is shown before the species name. Abbreviations: BRZ: Brazil; MXC: Mexico; USA: United States of America; PAN: Panama; CHL: Chile.

The concatenated topology obtained for the 16S rRNA and COI genes (Fig. 4) recovered the main groups that were observed in the two separate analyses carried out for each gene. All specimens of *Emeritaalmeidai* sp. nov. were clustered together in a well-supported clade. The only specimen of *E.portoricensis* included in the analysis was well separated from other species. These two groups were recovered as sister species in a larger clade, as can be observed in the 16S rRNA and COI phylograms. *Emeritarathbunae* was recovered as the sister species of *E.brasiliensis*, and *E.talpoida* was recovered as the sister species of the clade composed by *E.rathbunae* and *E.brasiliensis*. The division of *E.talpoida* in two subclades was not observed because there were only two specimens of this species. However, subtle differences could be inferred by the long branches connecting the two specimens within this clade. *Emeritabenedicti* was recovered as the sister species of all other species of *Emerita* in the analysis.

## ﻿Taxonomy

**Figure 5.**
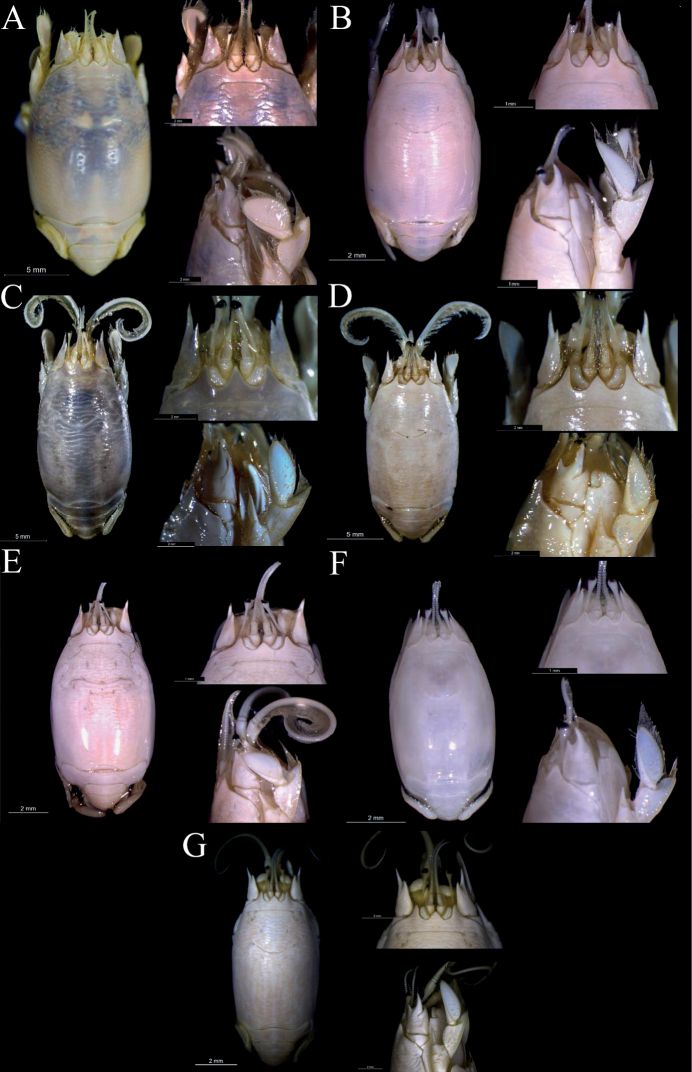
Dorsal view of carapace and rostrum/front, and lateral view of antenna/first pereopod of adult ♀s of *Emerita* species from the Americas **A***Emeritaanaloga* (CCDB 4870) **B***Emeritabenedicti* (CCDB 4674) **C***Emeritabrasiliensis* (CCDB 4615) **D***Emeritaalmeidai* sp. nov. (CCDB 3369) **E***Emeritarathbunae* (CCDB 1029) **F***Emeritatalpoida* (CCDB 4675) **G***E.portoricensis* (CCDB 3525).

**Figure 6.**
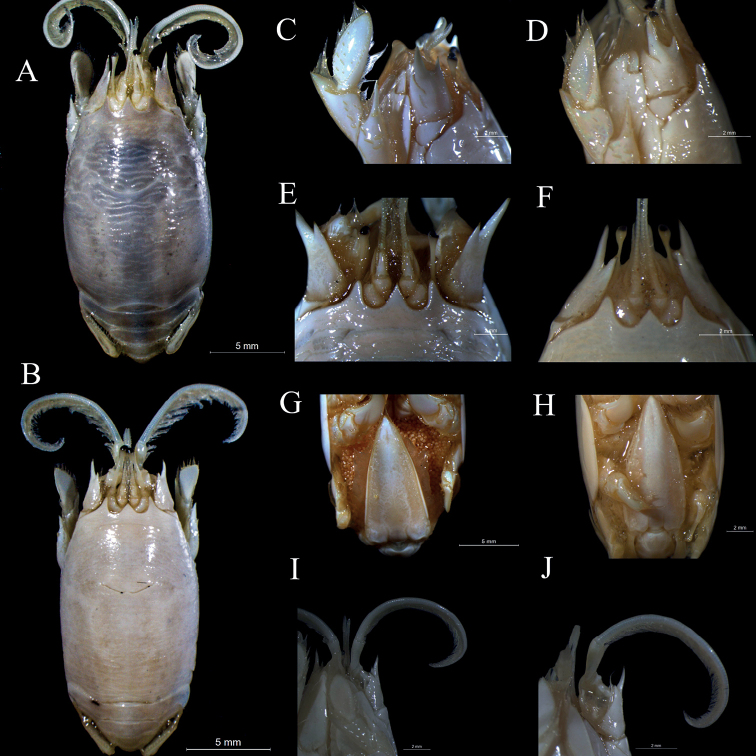
Comparative morphological characters observed in adult ♀s of similar sizes between *Emeritabrasiliensis* (CCDB 2552) and *E.almeidai* sp. nov. (CCDB 3026). Carapace shape: Dorsal view of **A***E.brasiliensis* and **B***E.almeidai* sp. nov.; Dactylus of first pereopod: Lateral view of **C***E.brasiliensis* and **D***E.almeidai* sp. nov.; Anterior region with rostrum, lateral spines, and ocular peduncle: Dorsal view of **E***E.brasiliensis* and **F***E.almeidai* sp. nov.; Posterior region/telson: Ventral view of **G***E.brasiliensis* and **H***E.almeidai* sp. nov.; Left ventral view of antenna **I***E.brasiliensis* and **J***E.almeidai* sp. nov.

**Figure 7.**
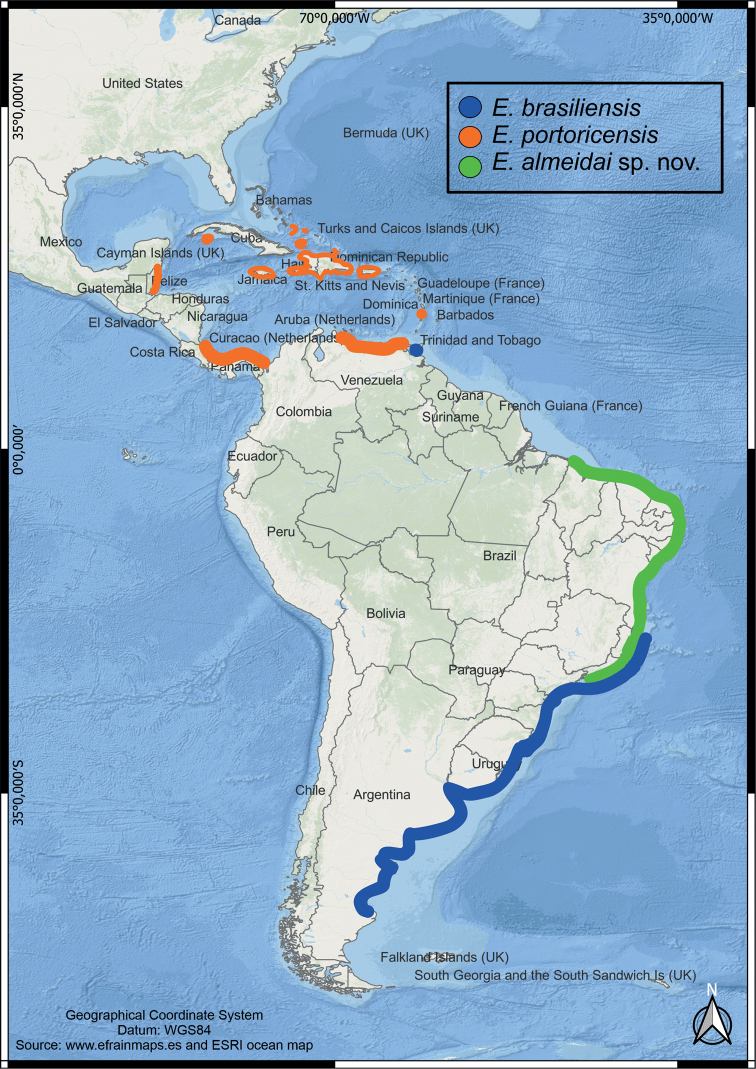
The updated geographic distribution of *Emeritabrasiliensis*, *E.portoricensis*, and *E.almeidai* sp. nov. according to the literature (Efford 1976; Melo 1999; Veloso and Cardoso 1999; Felder et al. 2023) and the present study.

Below we present the list of examined material and the description of the new species. A comparative image shows details about the general morphology of the seven species of *Emerita* from the Americas (Fig. 5) and a detailed comparison between *E.brasiliensis* and *E.almeidai* sp. nov. (Fig. 6) is furnished to complement the information. The updated distribution (Fig. 7) and a comparative analysis of the main characters of these two species and *E.portoricensis* was presented in Table 2.

**Table 2.** Diagnostic characters (Calado 1990; Melo 1999; Felder et al. 2023; present study) used for comparison between three studied species.

Characters	*E.brasiliensis*	*E.portoricensis*	*E.almeidai* sp. nov.
Antennal flagellum	103–134 articles	76–86 articles	74–104 articles
Ocular peduncle	Not exceeding the spines of the second antennal article	Usually extending beyond the spines of the second antennal article	Usually extending beyond the spines of the second antennal article
Front (rostrum + lateral projections)	Anterior margin with two triangular projections with rounded distal ends that are ca. the same size as the rostrum	Anterior margin with two triangular projections that extend beyond the level of the rostrum	Anterior margin with two triangular projections that extend beyond the level of the rostrum
Dactylus of first pereopod	Wide, oval shaped, inferior margin not serrated	Narrow, visibly longer than wide, inferior margin slightly, irregularly, or inconspicuously serrated	Narrow, visibly longer than wide, inferior margin conspicuously and regularly serrated
Carapace	Proportionally wider than that of *E.portoricensis* and *E.almeidai* sp. nov.	Proportionally longer than that of *E.brasiliensis* and *E.almeidai* sp. nov.	Proportionally longer than that of *E.brasiliensis*; proportionally wider than that of *E.portoricensis*
Carapace rugae	Broken into cusps	Dense and non-broken	Dense and non-broken
Telson	Distal end reaching the proximal region of the coxa of the first pereopod	Tends to be longer than wide in relation to the telson of *E.brasiliensis*	Tends to be longer than wide in relation to the telson of *E.brasiliensis*
Coloration	Brownish white or olive brown throughout	Olive brown carapace with wide white lines and markings, a white line marking posterior 1/4 of carapace, most rugae the same color as carapace, pleon with alternating olive brown and white bars	Olive brown carapace with slim white lines and markings, line marking posterior 1/4 of carapace usually absent, rugae white in color contrasting with carapace, pleon with alternating olive brown and white bars

### ﻿Superfamily Hippoidea Latreille, 1825

**Family Hippidae Latreille, 1825**

***Emerita* Scopoli, 1777**

#### ﻿ Emerita almeidai

Taxon classificationAnimaliaDecapodaHippidae

Mantelatto & Balbino sp. nov.

A9DEB82C-F5D1-59D0-8C89-F674B081CE48

https://zoobank.org/6A094FCB-F019-4853-B561-E427D0963CA8

Figs 1 , 5 , 6 , 8 , 9 , 10 , 11 , 12

Emerita portoricensis – Efford, 1976: 178, 179; Calado 1990: 266, 268, 271; Tam et al. 1996: 490; Haye et al. 2002: 904 (non Emeritaportoricensis Schmitt, 1935).

##### Type material.

***Holotype***: ovigerous ♀ (cl. 13.52 mm), CCDB 7233, Praia do Paiva (lower intertidal, quartzite, coarse sand off wave-washed beach), Ilha do Amor, Cabo de Santo Agostinho, PE, Brazil, 08°13'48"S, 34°55'22"W, 27 August 2022, colls. Mantelatto, F.L., Bochini, G.L., Balbino, F.C., Rios, A. ***Paratypes***: 3 ovigerous ♀s (cl. 17.31 mm, 17.93 mm, 15.90 mm), 1 ♀ (cl. 14.67mm) (1 ovigerous ♀ cl. 17.31 mm dissected – left antennule, antennae, mouthparts, maxillipeds, pereopods, uropods and telson), CCDB 5855, Praia de Serrambi, Município de Serrambi, Ipojuca, PE, Brazil, 08°33'39.91"S, 35°00'45.15"W, 20 July 2015, colls. Mantelatto, F.L., Mantelatto, F.B., Biagi, R.; 3 ovigerous ♀s (cl. 15.01 mm, 15.93 mm, 9.94 mm), 4 ♀s (cl. 9.66 mm, 9.64 mm, 9.24 mm, 9.02 mm), 1 juvenile (cl. 4.72 mm), “1 ovigerous ♀ (cl. 15.01 mm dissected – mouthparts, maxillipeds, pereopods, uropods and telson), CCDB 4937, Praia de Boa Viagem, Recife, PE, Brazil, 08°08'12.96"S, 34°54'05.84"W, 28 January 2014, colls. Mantelatto, F.L., Mantelatto, F.B., Biagi, R.; 1 ♀ (cl. 10.49 mm), MOUFPE 20112, Praia do Paiva, Ilha do Amor, Cabo de Santo Agostinho, PE, Brazil, 08°13'48"S, 34°55'22"W, 27 August 2022, colls. Mantelatto, F.L., Bochini, G.L., Balbino, F.C., Rios, A.; 1 ♂ (cl. 7.29 mm), MZUSP 43536, Praia do Forte Orange, Vila Velha, Ilha de Itamaracá, PE, Brazil, 07°50'40"S, 34°50'33"W, 30 August 2022, colls. Mantelatto, F.L., Bochini, G.L., Balbino, F.C., Rios, A., Almeida, A.O.”

##### Additional material.

1 ♀, CCDB 4526, Morro Branco (CE), 25 March 1989; 1 ♀, 1 ovigerous ♀, CCDB 3369, Praia de Perobas, Touros (RN), 10 June 2011, colls. Robles, R., Pileggi, L.G.; 5 ovigerous ♀s, CCDB 3376, Praia de Maracajaú, Maxaranguape (RN), 10 June 2011, colls. Robles, R., Pileggi, L.G.; 2 ♀s, 1 ovigerous ♀, CCDB 3393, Morro do Careca, Ponta Negra, Natal (RN), 06 June 2011, coll. Robles, R.; 1 ♀, 4 ovigerous ♀s, CCDB 3380, Morro do Careca, Ponta Negra, Natal (RN), 07 June 2011, coll. Robles, R.; 2 ♀s, 10 ovigerous ♀s, CCDB 4869, Praia de Maragogi, Maragogi (AL), 05 October 2013, colls. Mantelatto, F.L., Mantelatto F.B.; 2 ♀s, 2 ovigerous ♀s, CCDB 6127, Praia de Imbassaí, Mata de São João (BA), 25 January 2017, colls. Mantelatto, F.L., Mantelatto, F.B.; 1 ovigerous ♀, CCDB 2606, Praia do Pé da Serra, Uruçuca (BA), 31 March 2009, colls. Mantelatto, F.L., Almeida, A.O.; 1 ovigerous ♀, CCDB 2605, Praia do Sul, Km 01, Hotel Praia do Sol, Ilhéus (BA), 30 March 2009, colls. Mantelatto, F.L., Almeida, A.O.; 2 ovigerous ♀s, CCDB 3026, Praia do Sul, Km 01, Hotel Praia do Sol, Ilhéus (BA), 10 November 2010, colls. Mantelatto, F.L., Peiró, D.F.; 1 ♀, 1 ♂, CCDB 4262, Praia da Lagoa Pequena, Prado (BA), 12 August 2012, colls. Carvalho, F.L., Souza-Carvalho, E.A.; 1 ♂, 3 ♀s, 1 ovigerous ♀, CCDB 3992, Praia de Iriri, Iriri (ES), 19 June 2012, colls. Carvalho, F.L., Robles, R., Peiró, D.F.; 2 ovigerous ♀s, CCDB 4376, Pedra do Sal (RJ), 19 November 2009, coll. Arresda, E.

##### Comparative material.

*Emeritaanaloga*: 4 ovigerous ♀s, CCDB 4870, Calfuco, XIV Región, Chile, 20 August 2013, coll. Fuentes, J.P.; *Emeritabenedicti*: 5 ♀s, 4 juveniles, CCDB 4674, Playa Escondida, Los Tuxtlas, México, 07 February 2013, coll. Robles, R.; *Emeritabrasiliensis*: 7 ♀s, 6 ♂s, CCDB 3990, Laguna Marginal, Guarapari (ES), Brazil, 18 June 2012, colls. Carvalho, F.L., Robles, R., Peiró, D.; 7 ♂s, 12 ♀s, 1 ovigerous ♀, 5 juveniles, CCDB 7226, Praia de Iriri, Anchieta (ES), Brazil, 19 June 2012, colls. Carvalho, F.L., Peiró, D., Robles, R.; 3 ♀s, CCDB 1030, Ubatuba (SP), Brazil, 20 November 2002, colls. Mantelatto, F.L., Scelzo, M.A.; 1 ♀, 1 ovigerous ♀, CCDB 2552, Praia Juquehy, São Sebastião (SP), Brazil, 26 December 2008, colls. Mantelatto, F.L., Mantelatto, F.B., Biagi, R.; 1 ♀, CCDB 7301, Praia de Guaratuba, Bertioga (SP), Brazil, 07 January 2023, colls. Mantelatto, F.L., Mantelatto, F.B., Mantelatto, H.B.; 2 ♂s, 6 ♀s, 2 ovigerous ♀s, CCDB 3924, Praia Guaiuba, Guarujá (SP), 22 October 2011, colls. Rossi, N.; Leone, I., Carvalho, F.L., Costa, A.; 5 ♀s, CCDB 1443, Praia Itararé, São Vicente (SP), Brazil, 23 October 2011, colls. Rossi, N., Leone, I., Carvalho, F.L., Costa, A.; 3 ♂s, 1 ♀, 7 ovigerous ♀s, CCDB 4409, Praia de Balneário Camboriú, Camboriú (SC), Brazil, 04 December 2012, colls. Carvalho, F.L., Souza-Carvalho, E.A.; *Emeritaportoricensis*: ovigerous ♀ (holotype, USNM 65731, photos), Mayaguez, Puerto Rico, 19–20 January 1899; 3 ♀s, CCDB 3525, Playa Boca Del Drago, Bocas Del Toro, Panamá, 06 August 2011, colls. Mantelatto, F.L., Negri, M.P., Rossi, N., Magalhães, T.; *Emeritarathbunae*: 3 ♀s, CCDB 1029, Playa Del Revolcadero, Granjas Del Marquez, Acapulco, México, 06 May 2012, coll. Mantelatto, F.L.; *Emeritatalpoida*: 9 ♀s, CCDB 4675, Playa Escondida, Los Tuxtlas, México, 07 February 2013, coll. Robles, R.

##### Diagnosis.

Carapace dorsally convex, 1.42–1.54× longer than wide, surface densely covered by microcrenulate rugae; most rugae elongate and continuous across carapace median line not forming rows or lines; 17 or more rugae crossing median line, rugae obsolete laterally on epimeral lobes. Front with three distinct subacute lobes consisting of rostrum and two lateral projections, rostrum visibly shorter than lateral projections. Antennular flagellum dorsal ramus with 30 articles. Antennal peduncle second article large, with three distal spines, median spine the longest, antennal flagellum with 74–104 articles. First maxilla proximal endite rounded, subcircular with margins visibly convex; endopodal palp wide, short, distal end upturned. Third maxilliped without exopod, endopod with merus distal inner margin projected into strong subtriangular lobe, lateral margins of merus sinuous, outer distal margin ending on acute angle. First pereopod merus large, inflated, broad truncate lobe on inferior margin of merus, carpus distal end with large spine, propodus ca. as long as dactylus; dactylus elongate, more than twice as long as wide, superior surface almost straight, inferior surface convex with low, moderate, and regularly spaced serrations, dactylus lined by long plumose setae and short spiniform setae or spinules, terminus of dactylus with single short spine, terminus subacute. Pleon with second pleonite larger than others, tergite as wide as carapace, sides of second pleonite forming wide flanges laterally, second and third pleonite with two pairs of rugae extending from junction with next pleonite almost to ventrolateral margin. Overall coloration olive grey, white laterally, rugae distinctly white in coloration, few thin white bars or stripes near posterolateral regions of carapace.

##### Description.

**Figure 8.**
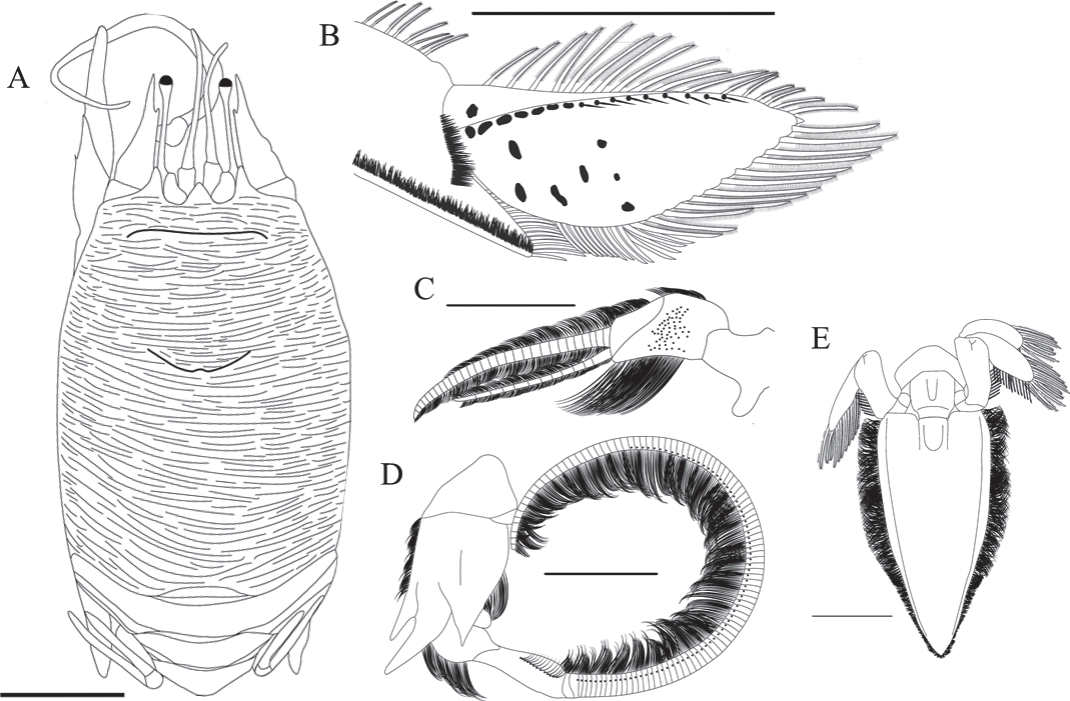
*Emeritaalmeidai* sp. nov. **A** ♀ paratype, cl. 14.67 mm (CCDB 5855) **B, E** ovigerous ♀ paratype, cl. 15.01 mm (CCDB 4937) **C, D** ovigerous ♀ paratype, cl. 17.31 mm (CCDB 5855) **A** dorsal view **B** lateral view of dactylus of pereopod 1 **C** lateral view of left antennule **D** lateral view of left antenna **E** dorsal view of telson and uropods. Scale bars: 6 mm (**A**); 4 mm (**B, E**); 3 mm (**C, D**).

***Carapace*** (Figs 1A, 5D, 6B, 8A, 11, 12A) elongate, 1.42–1.54× longer than wide, subcylindrical, overall dorsally convex, highly convex transversely, slightly convex longitudinally; carapace surface densely covered by low transverse microcrenulate to microdenticulate rugae, many of which are continuously elongate, not forming proper lines or rows of rugae, many continuous across middorsal region on anterior and posterior portions of carapace, usually 17 or more rugae extending across postcervical middorsal line; pterygostomial region with ventrolateral rugae; rugae separating small, anteriorly curved ridges; anterior margin of broad epimeral lobe of carapace with serrated appearance due to presence of such ridges. Pterygostomial plates densely punctate, separated from carapace by post-gastric groove; low slightly rugose ridge extending from median portion almost to distal end of plate, parallel to carapace margin for most of its extension, slightly deflected inwards near distal end. Front (Figs 5D, 6F) with three subacute dentiform projections; median projection forming broad triangular or subtriangular rostrum surrounded by relatively long plumose setae, distal end of rostrum sharply pointed, rostrum visibly shorter than lateral projections; lateral projections subtriangular with concave sides proximally and straight sides distally, visibly longer than rostrum, also surrounded by relatively long plumose setae; rostrum and lateral projections separated by wide U-shaped sulcus. Anterolateral margins of carapace just to the side of frontal projections surrounded by short plumose setae. Transverse frontal groove parallel to front, mostly straight, slightly bent at lateral extremes. Cervical groove just anterior to midlength of carapace, crescent shaped with convex face facing posteriorly, slight anteriorly facing notch on cervical groove on carapace midline. Most rugae broken, obsolete or absent on lower broad epimeral lobe.

***Eyes*** (Fig. 5D) swollen at end of very narrow and elongated peduncles, reaching anteriorly past distal portion of fifth antennal peduncle article when extended and past spines of second antennal article when retracted; ocular peduncles composed of three articles; first article arcuate, longer than wide, convex on internal face and concave on external face; second article deflected downwards, longer than first article; third article long, first third wider, other two thirds very narrow, widening near eye.

***Antennules*** (Fig. 8C) short; antennular peduncle composed of three articles; first article wider than others, external surface with large dentiform projection near base of article; second article densely setose, trapezoid in shape, dorsal surface shorter, ventral surface longer; third article short, also trapezoid in shape, dorsal surface longer, ventral surface shorter; flagellum dorsal ramus longer, with 30 articles, ventral ramus shorter, with 12 articles.

***Antenna*** (Figs 6J, 8D) long; antennal peduncle composed of five articles; first article trapezoidal, longer than wide; second article large, covered by sparse rugae, distal end with three large spiniform projections, median projection longest, dorsal and ventral projections ca. the same size as each other, sulcus extending across dorsolateral surface from proximal end to base of dorsal spiniform projection, microdenticulate ridge separating ventral projection from median projection, one row of setose rugae present on mesial ventral portion; third article inserted on lateral portion of second article, completely concealed by second article in lateral view, trapezoidal in shape, proximal portion rectangular in shape, distal portion triangular, short line of setae parallel to distal margin; fourth article dorsally convex, ventrally Y-shaped; fifth article elongate, slimmer near base, inflated distally, row of setae on ventral margin; flagellum long, composed of 74–104 articles with dense long setae ventrally in adult specimens, number of articles smaller in juveniles, first article longest, ~ 3× as long as other articles.

**Figure 9.**
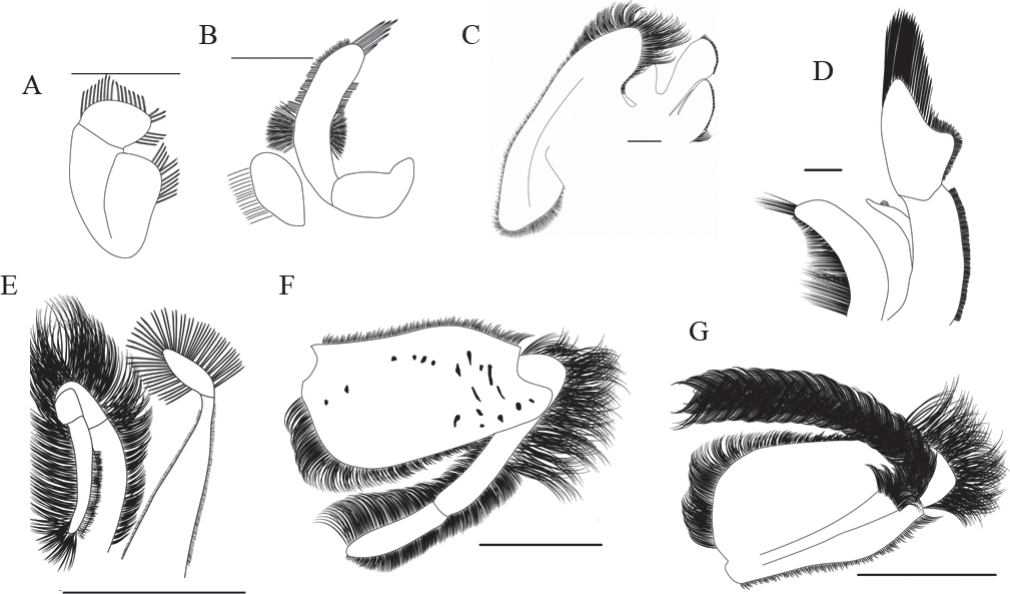
*Emeritaalmeidai* sp. nov. **A–E** ovigerous ♀ paratype, cl. 17.31mm (CCDB 5855) **F, G** ovigerous ♀ paratype, cl. 15.01 mm (CCDB 4937) **A** right mandible **B** left first maxilla **C** right second maxilla **D** left first maxilliped **E** left second maxilliped **F** right third maxilliped external face **G** right third maxilliped internal face. Scale bars: 1 mm (**A–D**); 4 mm (**E–G**).

***Mandible*** (Fig. 9A) membranous, mostly fused with posterior margin of epistome; gnathal lobe short, kidney-shaped, inserted basally on mandible, projected inwards, external margin convex, internal margin concave, long plumose setae along mesial and distal margins; palpus composed of two articles, longer than gnathal lobe; first article subrectangular; terminal article suboval, lined by many long setae.

***First maxilla*** (Fig. 9B) small; proximal endite loosely connected to rest of appendage, oval, flattened, lateral and distal margins surrounded by relatively long setae; distal endite elongate, narrow, distal end slightly wider, pin-shaped, margins lined by setae, setae on proximal internal side very long, median and distal setae shorter, setae on proximal and median external portions shorter, longer subdistally; endopodal palp nearly as wide as long, tip slightly hooked upwards.

***Second maxilla*** (Fig. 9C) exopod developed as scaphognathite attached to base, proximal and distal lobes flattened, lined by long setae, proximal lobe semi-oval in shape, broader, distal lobe semi-oval, slimmer; endopod short, wider proximally, narrowed towards distal end, subacute tip deflected distally.

***First maxilliped*** (Fig. 9D) membranous; exopod larger, arched, composed of two articles; proximal article subrectangular, outer margin convex, inner margin concave, distal article subovoid, surrounded distally by many long plumose setae, outer margin proximally convex until ca. midpoint, where it becomes concave, inner margin convex throughout its extension; endopod minute, elongate, membranous, with small tuft of setae subterminally; distal endite crescent shaped, exceeding length of exopod first article, extensively covered by short setae on external surface, inner margin covered by dense long plumose setae, patch of relatively long plumose setae present on distal end.

***Second maxilliped*** (Fig. 9E) membranous, exopod and endopod subequal; exopod composed of two articles; proximal article elongate, subtriangular, widest proximally, narrowing towards distal end, sparsely setose; distal article subovoid, margins surrounded by long plumose setae; endopod composed of four articles; ischio-merus narrow, arcuate in shape, proximal end wider, narrowing towards distal end, outer margin convex lined by several long setae, inner margin concave lined by several setae; carpus short, inflated, distal and lateral margins densely covered by long plumose setae; propodus short, less robust than carpus, margins covered by setae; dactylus long, narrow, elongate, widest at base, narrowing towards distal rounded end, ~ 2/3 as long as ischio-merus, surrounded by long setae.

***Third maxilliped*** (Fig. 9F, G) lacking exopod; endopod with coxa wider than long, small subtriangular projection on proximal inner side, base-ischium minute, much wider than long, merus broad, sparse setose rugae present on outer face, inner face mostly smooth with one large and distinct ridge crossing merus from base-ischium junction to carpus junction, margins lined by short setae, long plumose setae distally, nearly twice as long as wide, proximal third of inner margin very rounded, convex, distal two thirds straight, large subtriangular projection on distal end of inner portion of merus overlying part of carpus and propodus, outer margin slightly concave proximally, convex distally, distal end of outer portion of merus straight, carpus short, subquadrate, distal margin and inner face covered by long setae, propodus long, slightly curved inwards, outer face smooth, inner face densely covered in setae, dactylus long, shorter than propodus, slightly curved inwards, distal end rounded, outer face smooth inner face densely covered in setae.

**Figure 10.**
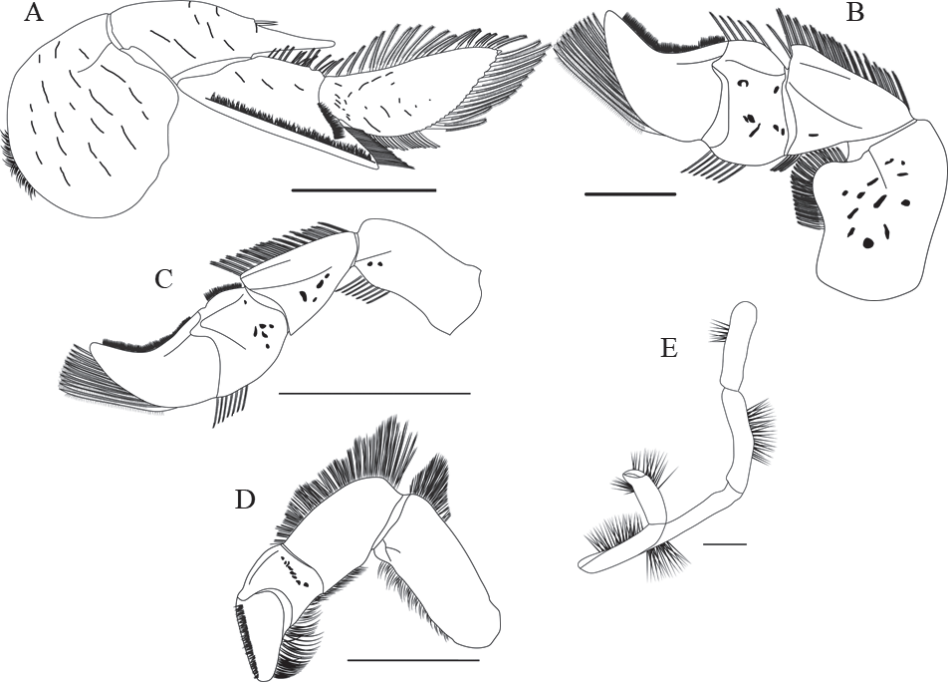
*Emeritaalmeidai* sp. nov. **A, D** ovigerous ♀ paratype, cl. 17.31 mm (CCDB 5855) **B, C, E** ovigerous ♀ paratype, cl. 15.01 mm (CCDB 4937) **A** lateral view of right pereopod 1 **B** lateral view of left pereopod 2 **C** lateral view of left pereopod 3 **D** lateral view of left pereopod 4 **E** lateral view of right pereopod 5. Scale bars: 4 mm (**A**); 2 mm (**B**); 5 mm (**C**); 3 mm (**D**); 1 mm (**E**).

***First pereopod*** (Fig. 10A) coxa subtrapezoidal, sparsely covered by setose rugae on external face, setae on ventral margin and distal end, ventral dentiform projection proximally; base-ischium longer than wide, minute, lined by setae ventrally, with two lobes separated by median notch, ventral margin surrounded by setae; merus large, subcircular, sparsely covered by setose rugae, superior margin convex, inferior margin convex proximally, extended laterally forming truncate lobe with straight or almost straight lateral margin, small short sulcus on mesial portion of distal end of merus; carpus elongate, crossed by some oblique rows of setose rugae on distal ventral portion, three very distinct small perpendicular rows of setose rugae on dorsal surface separating article from large narrow distal spine, spine reaching to base of dactylus; propodus subtrapezoidal in shape, sparsely surrounded by setae, sparse setose rugae present, a transversal ridge running along most of ventrolateral portion of propodus, including distal process (Fig. 8B), dense short setae running along ridge, distal process long, subtriangular, strong oblique ridge running from dactylus junction to base of distal process, superolateral surface of distal process excavate, fits base of dactylus, long setae present on distal process; dactylus (Figs 5D, 6D, 8B) elongate, usually more than twice as long as wide, superior margin mostly straight, inferior surface convex, inferior surface moderately serrated from median portion to distal end, terminus of dactylus acute or subacute bearing one small spine, weakly arched oblique ridge across superior portion of dactylus, ridge originating near median portion of junction with propodus, running upwards towards superior margin, fusing with superior margin around median portion of dactylus, ridge lined by small setae, long plumose setae surrounding dactylus, small spiniform setae among them.

***Second through fourth pereopods*** (Fig. 10B, C, D) similar in configuration. Second pereopod coxa subquadrate; base-ischium small, longer than wide, surrounded by setae; merus large, subrectangular, longer than wide, surface covered by sparse setose rugae, superior margin mostly straight, convex towards distal end, inferior margin slightly concave, large dentiform projection protruding ventrally from distal end of merus, inferior margin lined by setae; carpus subtriangular, external surface with two short ridges present, one on superior and one on inferior regions of article, internal surface setose, crossed mesially by single row of setae, inferior portion with small triangular projection distally lined by setae; propodus wider than long, subrectangular, superior margin oblique, external face with small triangular projection positioned distally on dorsal region overlying part of dactylus, transverse ridge near superior margin, internal face with large spiniform projection lined by large setae at ca. same position; dactylus large, flattened, hook-shaped, broad proximally, narrowing distally, superior margin concave, inferior margin convex, distal tip upturned, inferior margin surrounded by long setae. Third pereopod coxa and base-ischium similar to second pereopod; merus subrectangular, much longer than wide; carpus very large, subtriangular, superior and inferior ridges present, propodus wider than long, subrectangular, superior margin oblique, small ridge near superior margin present, small triangular projection positioned distally on superior margin overlying part of dactylus; dactylus large, hook-shaped, flattened, broad proximally, narrowing distally, superior margin concave, inferior margin convex, distal tip upturned, inferior margin surrounded by setae. Fourth pereopod coxa and base-ischium similar to that of second and third pereopods; merus elongate, much longer than wide, subrectangular; carpus large, longer than wide, inferior surface almost straight, superior surface convex; propodus subquadrate, nearly as wide as long, lacking triangular projection, line of short setae near superior margin; dactylus large, somewhat flattened, broad distally, narrowing towards distal end, proportionally smaller than in other pereopods, subtriangular, superior and inferior margins almost straight, tip not upturned, a line of short setae parallel to superior margin, inferior margin lined by setae.

***Fifth pereopod*** (Fig. 10E) reduced, concealed under carapace; all articles except for dactylus elongate, much longer than wide, with small tufts of setae distally; propodus long, with distal projection that along with dactylus forms a small chela; dactylus short, deflected inwards; chela small, covered by setae.

***Pleon*** short, partly recurved under carapace. First pleonite smallest, minute, much wider than long, fitting into posterior concavity of carapace; second pleonite larger than others, as wide as carapace, median portion of pleonite narrow, both sides of pleonite enlarged, forming two wide lateral flanges, flanges with pair of long transverse rugae extending from third pleonite junction almost to ventrolateral margins of tergite, distal portion of ventrolateral region of each pleonite with short transverse ruga extending from superior margin to inferior margin of narrowest portion of flange, wide lateral flanges forming space where third pleonite fits; third pleonite smaller than second, sides of pleonite somewhat enlarged forming flanges that are mostly covered by flanges of second pleonite, two transverse rugae extending from junction with fourth pleonite to junction with second on each flange; fourth pleonite smaller than third, sides slightly enlarged forming flanges which are mostly covered by flanges of third pleonite, one oblique ruga extending from junction with fifth pleonite to junction with third on each side of pleonite; fifth pleonite smaller than fourth, lateral flanges small; sixth pleonite subpentagonal, lateral margins forming subtriangular projections, two short longitudinal grooves near articulation with telson, each groove joined to two much smaller transverse grooves. Female pleopods on second through fourth pleonites developed as three long and narrow articles, not developed on first and fifth pleonites; males without developed pleopods on first through fifth pleonites; uropods large, protopod subrectangular, endopod suboval, rounded, distal margin densely covered in setae, exopod suboval, more elongate, distal margin densely covered in setae.

***Telson*** (Figs 6H, 8E) lanceolate, lateral margins setose, slightly convex proximally, very slight notches at ~ 3/4 of length of telson, two short longitudinal grooves near junction with pleon, two long longitudinal ridges parallel to lateral margins of telson, distal end of telson subacute.

##### Coloration in life.

Carapace overall olive grey dorsally, lateral regions white, rugae extending across carapace white, posterolateral regions of carapace with few slim white longitudinal lines or small white blotches; lines and blotches usually restricted to posterolateral region, but some specimens possess one white longitudinal line along posterior 1/4 of carapace median line. Pleonal somites olive-grey anteriorly, white posteriorly, forming a pattern of alternating olive-grey and white stripes (Fig. 12A).

##### Habitat.

Shallow infaunal, lives in wave swash zone of sandy beaches or shallow subtidal sandy flats where it burrows shallowly in sand, moves with tidal rise and fall.

##### Distribution.

Brazil: known from Maranhão, Ceará, Rio Grande do Norte, Paraíba, Pernambuco, Alagoas, Sergipe, Bahia, Espírito Santo, and Rio de Janeiro.

##### Etymology.

The species name honors Alexandre O. Almeida, a valued friend and respected colleague who has contributed extensively to increase knowledge of the decapod crustaceans of Brazil.

##### Remarks.

**Figure 11.**
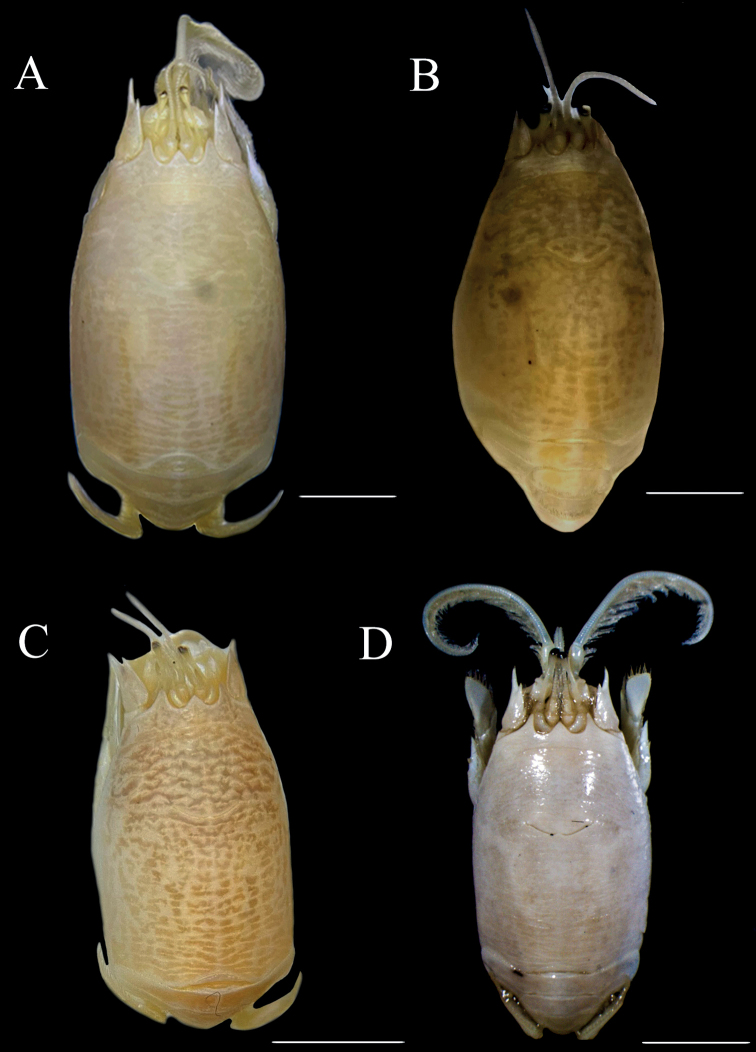
*Emeritaalmeidai* sp. nov. **A** ovigerous ♀ holotype, cl. 13.52 mm (CCDB 7233) **B** ♂ paratype, cl. 7.29 mm (MZUSP 43536) **C** ♀ paratype, cl. 10.49 mm (MOUFPE 20112) **D** ♀, cl. 10.20 mm (CCDB 3369). Scale bars: 4 mm (**A, B**); 2 mm (**C**); 5 mm (**D**).

**Figure 12.**
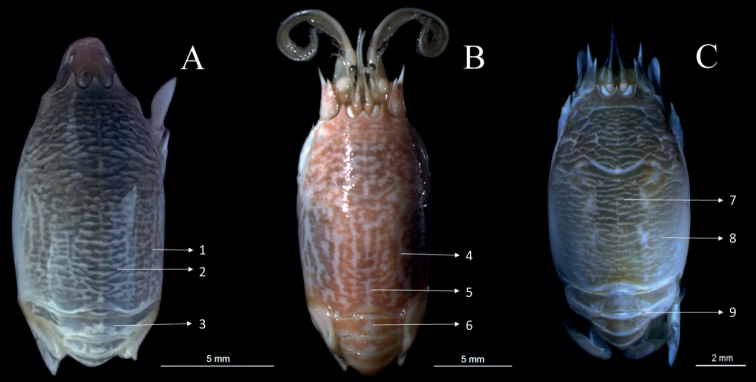
Fresh coloration of *Emeritaalmeidai* sp. nov., *E.portoricensis* and *E.brasiliensis***A***E.almeidai* sp. nov., ovigerous ♀ (not deposited), live specimen, Porto de Galinhas, PE, Brazil **B***E.portoricensis*, ♀ (CCDB 3525), freshly collected specimen, Boca del Drago, Panama **C***Emeritabrasiliensis*, ♀ (CCDB 7301), freshly collected specimen, Praia de Guaratuba, Bertioga (SP), Brazil. Coloring details: 1. Thin white stripes on posterolateral portions of carapace; 2. Rugae clearly surrounded by white coloration; 3. Striped pattern on pleon; 4. Wide white bars on posterolateral portions of carapace; 5. White bar along posterior 1/4^th^ of median line of carapace; 6. Striped pattern on pleon; 7. Olive brown or brownish white color overall; 8. Rugae broken into cusps; 9. Absence of striped pattern on pleon.

*Emeritaalmeidai* sp. nov. is closest to *E.portoricensis* and thus shares a wide range of morphological similarities, which is why for many years several specimens from Brazil were wrongly assigned to *E.portoricensis* (see Introduction). Both species have a carapace densely covered by microcrenulate rugae (Figs 1A, 5D, G, 6B, 8A, 11, 12) distributed in similar patterns, a front with three subacute lobes with the rostrum being distinctly shorter than lateral projections (Figs 5D, G, 6B), first pereopod dactyli more than twice as long as wide and not as rounded as in other species such as *Emeritabrasiliensis* and *Emeritatalpoida* (Figs 5, 6C, D), two pairs of rugae extending onto lateral flanges of the first two pleonites. However, some characters such as the carapace length and width ratio (cw./cl.), the number of articles on the antennal flagellum, the first maxilla, the dactylus of the first pereopod, and the coloration in life can be used to distinguish between these two species. The carapace in *E.almeidai* sp. nov. (Figs 1A, 5D, 6B, 8A, 11, 12A) tends to be more oblong than that of *E.portoricensis* (Figs 5G, 12B), usually being 1.42–1.54× as wide as long in adult specimens (vs. 1.49–1.64× in *E.portoricensis*, present study), although there is some overlap. The number of articles on the antennal flagellum of *E.almeidai* sp. nov. (Fig. 8D) also tends to be more variable than that of *E.portoricensis*, varying from 74 to 104 articles in adult specimens, while *E.portoricensis* usually has 76–86 (Felder et al. 2023; present study). Although there is still some overlap, this character is still useful to distinguish between the two species. However, juvenile specimens (see details in Materials and methods) may have many fewer articles on the antennal flagellum in both species, and thus this character is only useful for adult specimens. The first maxilla of *E.almeidai* sp. nov. (Fig. 9B) also differs from that of *E.portoricensis* (Felder et al. 2023: 349, fig. 3): in *E.almeidai* sp. nov. the proximal endite is wider, rounder and with more convex margins, and the endopodal palp is proportionally wider and shorter. The first pereopod dactylus (Figs 5D, 6D, 8B) is also distinct, with *E.almeidai* sp. nov. having low, moderately and regularly spaced serrations on the inferior surface of the dactylus, while *E.portoricensis* has a slight and irregular serrations, which in many cases can be absent. The coloration of these two species can also be used to distinguish between live specimens. As shown in a recent redescription of *E.portoricensis* (Felder et al. 2023: 345, fig. 1) and in the present work (Fig. 12B), this species has very wide white bars on the posterolateral regions of carapace along with a wide white bar along the posterior 1/4 of the carapace median line. Although *E.almeidai* sp. nov. shares some of these characteristics (Fig. 12A), the white bars are usually slimmer and the white bar along the carapace median line is usually absent; however, it was observed only in one freshly collected paratype specimen (MZUSP 43536). The white colored rugae, which were observed in all of the freshly collected specimens of *E.almeidai* sp. nov. (Fig. 12A), however, are not present in either of the specimens shown in the recent redescription of *E.portoricensis* (Felder et al. 2023: 345, fig. 1) or in the specimen analyzed in this study (Fig. 12B), suggesting that this character might be unique to *E.almeidai* sp. nov. The southernmost record for *E.portoricensis* (Venezuela and Trinidad) and the northernmost record for *E.almeidai* sp. nov. (Maranhão, Brazil) are very far apart and there is a strong marine barrier, the Amazon–Orinoco plume (see Curtin 1986 for physical characteristics) that can promote some isolation between northern and southern decapod populations (see Peres et al. 2022), and this is possibly the reason there are no records of these species coexisting in the same environment.

*Emeritaalmeidai* sp. nov. has been observed to co-occur with *E.brasiliensis* in Praia de Iriri, in the state of Espírito Santo, Brazil (CCDB 3992 and 7226), with specimens of both species being collected at the same locality and on the same day. The distribution of these two species overlaps along the coast of the states of Espírito Santo and Rio de Janeiro, and it is possible that they co-occur in more locations in these states. *Emeritaalmeidai* sp. nov. can be distinguished from *E.brasiliensis* by the shape of the dactylus, which is elongated and has a serrated ventral margin in *E.almeidai* sp. nov. (Figs 5D, 6D, 8B) and ovate and non-serrated in *E.brasiliensis* (Figs 5C, 6C). The dactylus length and width ratio (dl./dw.) is also a robust parameter to distinguish the two species, especially in individuals of similar size. In *E.almeidai* sp. nov., the dactylus is proportionally longer and narrower than that of *E.brasiliensis*. The front is also different (Figs 5C, D, 6C, D): in *E.brasiliensis* the lateral projections and the rostrum are ca. as long as each other. The patterns of distribution of the microcrenulate rugae are also distinct between these two species (Figs 5C, D, 6A, B), with *E.almeidai* sp. nov. having very dense and non-broken rugae across much of the carapace, while *E.brasiliensis* has rugae that are more broken into cusps (Felder et al. 2023). Furthermore, the cw./cl. ratio is also useful to distinguish these two species since *E.almeidai* sp. nov. has an overall longer and narrower carapace when compared to *E.brasiliensis*. The antennae (Figs 6I, J, 8D) is another character that can be used to distinguish between the two species, because *E.almeidai* sp. nov. has 74–104 articles, while *E.brasiliensis* has 103–134 articles; however, there is a small overlap. The differences between the telson measurements obtained between the telson length and width ratio (tl./tw.) showed a tendency for telson growth in *E.almeidai* sp. nov. in relation to the increase in carapace length, while in *E.brasiliensis* this ratio tends to remain stable with increasing carapace length. Melo (1999) did not highlight significant differences for this structure, but Calado (1990) described the telson of *E.brasiliensis* as being lanceolate, larger than the pleon, with all margins with short bristles, and that of *E.almeidai* sp. nov. with a triangular shape, larger than the pleon, wider in the proximal portion, with margins also supporting short bristles, which corroborates the biometric data found in our analyses.

The other species of *Emerita* found in the western Atlantic Ocean, *E.talpoida* and *E.benedicti*, are not known to co-occur with *E.almeidai* sp. nov.; *Emeritatalpoida* can be distinguished from the new species by the rounded and ovate dactylus of the first pereopod (Fig. 5D, F), while *E.benedicti* (Fig. 5B) has a very acute terminus of the dactylus compared to a more subacute and slightly rounded terminus for *E.almeidai* sp. nov. The morphology of the first pereopod dactylus can also be used to distinguish the new species from other congeners in the Indian Ocean, Indo-Pacific and eastern Pacific.

Previous descriptions of mouthparts of species of *Emerita* are scarce, only existing for two species, *E.talpoida* and *E.portoricensis* (see Snodgrass 1952 and Felder et al. 2023). Thus, comparative studies of the mouthpart morphology of *Emerita* are lacking and could be of great importance. At least for *E.almeidai* sp. nov. and *E.portoricensis*, it has been noted that the morphology of certain articles of the mouthparts, in this case the first maxilla, can be successfully used to distinguish between species. Thus, future descriptions and redescriptions of species of *Emerita* should include such characters, which might be valuable for comparative taxonomic studies.

The number of species in the genus *Emerita* is now raised to 12, with five occurring in the western Atlantic (*E.almeidai* sp. nov., *E.benedicti*, *E.brasiliensis*, *E.portoricensis*, *E.talpoida*), five in the Indian Ocean or Indo-Pacific (*E.austroafricana*, *E.emeritus*, *E.holthuisi*, *E.karachiensis*, *E.taiwanensis*) and two in the Eastern Pacific (*E.analoga* and *E.rathbunae*). The actual number might even be higher, given that there is a large distribution hiatus between the populations of *E.analoga* from North and South America and genetic differences between the northern and southern populations of *E.talpoida*. The record of *E.brasiliensis* from Venezuela is doubtful and may be a misidentification or may represent a separate species given the large geographic hiatus (Fig. 7). All of these cases require a thorough study, as made herein for some congeners, to determine whether these actually represent different, yet very similar, species.

## ﻿Discussion

The combination of morphological and molecular methods confirms the validity of each species of *Emerita* included in the analysis and showed a clear division of *E.talpoida* into two subgroups that should be studied in the future. In addition, our phylogenetic trees based on two molecular markers confirmed the presence of a cryptic species previously misidentified as *E.portoricensis*, which we described in detail as *E.almeidai* sp. nov. For more than eight decades, a group of Brazilian specimens of *Emerita* was treated as *E.portoricensis* by several authors (see Introduction). The distribution of *E.almeidai* sp. nov. from Maranhão to Rio de Janeiro (Brazil) in combination with the redescription of *E.portoricensis* by Felder et al. (2023) answered questions that were raised in the past by some authors (Efford 1976; Calado 1990; Melo 1999) about the gap/discontinuity that existed in the distribution of *E.portoricensis*. These questions were clarified by the recognition of two different species, one in each hemisphere along the western Atlantic.

Species in the genus *Emerita* are known to have relatively long planktonic larval stages (i.e., *E.talpoida* lasting 30 days and *E.rathbunae* lasting 90 days, Efford 1970; *E.holthuisi* lasting 52 days, Siddiqi and Ghory 2006), thus favoring the chances of dispersion to suitable habitats. This extended larval development plays a critical role in governing the genetic structure, phylogeography, and dispersion of mole crabs of the genus *Emerita* (Dawson et al. 2011), factors which are also influenced and defined by other influences such as currents, transport effects, and sea level changes as observed in other groups (Doherty et al. 1995; Bohonak 1999; Cowen et al. 2000; Dawson 2001; Byers and Pringle 2006; Lester et al. 2007; Lessios 2008). Although the dispersal potential is high for species that present this larval profile (Palumbi and Benzie 1991), it does not always lead to strong gene flow, since physical or biological barriers can interfere with this dispersal process. Thus, disjunct populations can accumulate substantial genetic differences over time (Tam et al. 1996) that can result in the appearance of species that are not recognized as such due to the absence of studies covering all the different populations within the area of distribution as well as the lack of a representative set of specimens from these populations. The newly described species *E.almeidai* sp. nov. fits within this pattern, since it is a southern population that appears to be separated geographically by the Amazon–Orinoco plume, which has been shown to be an important physiological barrier for larval dispersal of many marine taxa, including decapod crustaceans (see Mandai et al. 2018 and Peres et al. 2022 for references and details).

There are some examples to support this hypothesis of separation for marine decapod crustaceans with a wide distribution along the western Atlantic, as noted in *E.almeidai* sp. nov. Using shrimps as an example, a recent study expanded the diversity of seabob shrimps of the genus *Xiphopenaeus* Smith, 1869 with descriptions of two new species (Carvalho-Batista et al. 2019), and a new species of *Latreutes* Stimpson, 1860 was described for a population from Brazil (Terossi et al. 2019). Considering the low number of integrative studies devoted to the huge diversity of decapods in the western Atlantic, these cases might not be exceptional, and we expect that a considerable number of cryptic and undescribed species may be revealed in the future.

### ﻿Insights on the evolution of the genus

There are no known fossils that provide information about the evolutionary history of *Emerita* or the paleontological origins of the genus. Molecular clock-based studies have suggested that all species of the genus evolved before the mid- to late Pliocene, although no centers of origin or biogeographic scenarios have been suggested. The hypothesis that *Emerita* species evolved at least before the late Neogene was raised by Tam et al. (1996). Vicariance and dispersal events probably played an important role in the speciation of *Emerita*. Other ecological, physiological, and oceanographic processes likely contributed to the final geographic distribution of the populations that gave rise to the different species of the genus seen today. The expansion and colonization of new geographic areas with subsequent reduction of gene flow were probably the mechanisms by which most of these species originated (Haye et al. 2002).

Species of *Emerita* present a geographic distribution in disjunctive regions, apparently with separate conspecific populations and/or with species that may coexist (see Tam et al. 1996 and Felder et al. 2023 for reviews). In the Americas, *Emeritaanaloga* is one of two species that inhabit the Pacific coast, recorded in both hemispheres, while *Emeritarathbunae* is restricted to the tropical region. In the western Atlantic, *Emeritatalpoida* is found from Massachusetts to Florida and also in the Gulf of Mexico; *Emeritabenedicti* occurs mainly in the inner part of the Gulf of Mexico; *Emeritaportoricensis* inhabits the tropical sandy islands and Central American mainland shorelines of the Caribbean Sea; *Emeritabrasiliensis* is distributed along the coast of southern South America, and *Emeritaalmeidai* sp. nov. is endemic to the Brazilian coast. These examples demonstrate that the vast majority of these species have wide geographic ranges and, thus, disjunct populations can be naturally genetically isolated (Tam et al. 1996), especially when natural barriers are present.

Outside of the Americas, *Emeritaholthuisi* has a very wide distribution, from the easternmost part of Africa to the southernmost part of India. It can also occur along the east coast of Africa, but so far there are few records for this region. *Emeritaemeritus* overlaps with *E.holthuisi* along the western coast of southern India and also occurs in the Indo-Pacific on the eastern coast of India, Malaysia, Indonesia (Efford 1976), and possibly in between. In the present study, these two species grouped together in a separate clade from the one composed of specimens from the Americas. *Emeritaaustroafricana* can be found along the southern portion of the east coast of Africa, in Mozambique, Madagascar, and South Africa (Schmitt 1937; Efford 1976). The molecular phylogenetic analysis using the COI gene carried out by Haye et al. (2002) recovered *E.austroafricana* as the sister species of *E.emeritus*, although *E.holthuisi*, the sister species of *E.emeritus* recovered in their phylogenetic analysis with the 16S rRNA gene and in the present study, was not included in the analysis. This suggests a close relationship between these three species, but the precise topology of the clade formed by these taxa cannot be determined at present. *Emeritakarachiensis* occurs on the west coast of the Indo-Pak subcontinent (Niazi and Haque 1974) and has not been included in any molecular analyses. However, Niazi and Haque (1974) mentioned morphological similarities between *E.karachiensis* and *E.holthuisi*, suggesting a close relationship between these species. Therefore, it is likely that this species is also part of the clade encompassing the Indo-Pacific species, although its exact position within this clade remains to be determined. *Emeritataiwanensis* is known only from two localities in Taiwan (Hsueh 2015) and has not been included in molecular phylogenetic analysis either. Hsueh (2015) suggested a close relationship between species that possess an acute and elongate pereopod 1 dactylus, such as *E.portoricensis*, *E.benedicti*, *E.holthuisi*, and *E.karachiensis*. However, as indicated by our phylogenetic analysis, this character does not seem to be phylogenetically informative, as it does not define any clades and appeared and was lost at least several times during the evolution of the species in the genus. Thus, the phylogenetic relationships of *E.taiwanensis* remain uncertain.

In the hypothesis proposed by Tam et al. (1996) on the evolution of the *Emerita* species from the Americas, it was suggested that *E.analoga* (Pacific species) was diverged from the other five New World species and was distant from *E.rathbunae* (the other Pacific species). Furthermore, *E.rathbunae* was clustered as sister species of the other species of *Emerita* found in the western Atlantic instead of with *E.analoga* that inhabits the Pacific coast. This hypothesis also suggests that *Emerita* species in the Americas evolved from an ancestral stock that was split into two branches, one leading to *E.analoga* and the other leading to the five remaining species. Our phylogenetic analysis corroborates this hypothesis, as *E.analoga* was recovered as the sister taxon to all other species of *Emerita*. Furthermore, it is likely that after splitting from *E.analoga*, the other populations of *Emerita* were divided into two groups, one that gave rise to the western Atlantic species and another that gave rise to the Indo-Pacific species. Consequently, with this scenario in mind, it can be assumed that the ornamentation of the second joint of the antennal peduncle present in *E.analoga* and some of the Indo-Pacific species (Schmitt 1937) is plesiomorphic, while the apomorphic condition is found in the clade composed of the other American species.

The biogeographic scenarios for the origin of *E.analoga* are not clear, and two hypotheses have been proposed (see references below): in the first, the genus *Emerita* originated on the western side of the Atlantic Ocean. If the center of origin is the Atlantic Ocean, it can be assumed that the species currently distributed in the eastern Pacific (*E.analoga* and *E.rathbunae*) evolved from Atlantic ancestors that dispersed into the Pacific and became isolated due to the closing of the Isthmus of Panama. The isthmus was closed to surface marine water circulation ~ 3 Mya (Late Neogene) but closed to deep-water circulation much earlier (Malfait and Dinkelman 1972; Keigwin 1982). In the second scenario, the center of origin of the genus *Emerita* was the Pacific Ocean. The Atlantic may have been colonized through the isthmus and taxa that differentiated in the Atlantic were likely to be ancestors of the species that later recolonized the Pacific, in this case *E.rathbunae* (Haye et al. 2002). At the end of the Cretaceous (~ 65 Mya), South America and Africa were completely separated (Dietz and Holden 1970). Although it is not possible to know how many species of *Emerita* inhabited the Tethys Sea before the complete separation of South America and Africa, it can be inferred that the separation of the continents was an important event in the speciation of these animals.

The clade composed of the northwest Atlantic species (*E.benedicti*, *E.talpoida*, *E.portoricensis*) and *E.almeidai* sp. nov. from the southwest Atlantic, as recovered in the 16S rRNA analysis, underwent extensive species diversification compared to the clade formed by *E.rathbunae* from the Pacific and *E.brasiliensis*, from the southwestern Atlantic, since the separation of the Pacific and Atlantic oceans ~ 3 Mya. This is consistent with other studies suggesting that the marine biota (mollusks, corals, and foraminiferans) of the western Atlantic were dramatically transformed ~ 2–3 Mya (Jackson and Budd 1996; Allmon 2001). Some hypotheses postulate that this change occurred as a result of the environmental disturbance associated with glaciation in the northern hemisphere and the formation of the Isthmus of Panama (Harrison 2004).

The closure of the Isthmus of Panama strongly affected ocean circulation, nutrient distribution, temperature, and salinity of the western Atlantic, and therefore had a significant influence on the evolution of marine fauna (Coates and Obando 1996; Allmon 2001). The flow of the Gulf Stream through the Yucatan Strait became more intense than it was before the closure of the Isthmus (Richards 1968) with an intense upwelling of cold deep waters (Stanley 1986). Therefore, this type of current may be a significant factor acting as a barrier to separate the group of species from the Gulf of Mexico from those located in the western side of the Atlantic, as observed in the *E.talpoida* clade, composed of specimens from Florida and Mexico in one group and individuals from Massachusetts and South Carolina in another group. There have been some suggestions of the occurrence of *E.talpoida* in Caribbean waters (see Felder et al. 2023, who say there is no confirmed evidence of this distribution). Previous usage of crabs as models of study (Felder and Staton 1994; Scheineider-Broussard et al. 1998) have identified the Florida Peninsula as a geographic barrier between the western Atlantic and Gulf of Mexico populations, raising awareness about the presence of cryptic species resulting from genetic isolation between populations distributed in these regions.

## Supplementary Material

XML Treatment for Emerita almeidai
